# Handgrip strength is associated with learning and verbal fluency in older men without dementia: insights from the NHANES

**DOI:** 10.1007/s11357-022-00703-3

**Published:** 2022-11-30

**Authors:** Konstantinos Prokopidis, Panagiotis Giannos, Theocharis Ispoglou, Ben Kirk, Oliver C. Witard, Yannis Dionyssiotis, David Scott, Helen Macpherson, Gustavo Duque, Masoud Isanejad

**Affiliations:** 1 Department of Musculoskeletal Biology, Institute of Life Course and Medical Sciences, University of Liverpool, Liverpool, UK; 2 Society of Meta-Research and Biomedical Innovation, London, UK; 3 Department of Life Sciences, Faculty of Natural Sciences, Imperial College London, London, UK; 4 Carnegie School of Sport, Leeds Beckett University, Leeds, UK; 5 Australian Institute for Musculoskeletal Science (AIMSS), The University of Melbourne and Western Health, St Albans, VIC 3021 Australia; 6 Department of Medicine-Western Health, Melbourne Medical School, The University of Melbourne, St Albans, VIC 3021 Australia; 7 Faculty of Life Sciences and Medicine, Centre for Human and Applied Physiological Sciences, King’s College London, London, UK; 8 Laboratory for Research of the Musculoskeletal System, National and Kapodistrian University of Athens, Kifissia, Greece; 9 1st Physical Medicine and Rehabilitation Department, National Rehabilitation Center EKA, Athens, Greece; 10 Institute for Physical Activity and Nutrition (IPAN), School of Exercise and Nutrition Sciences, Deakin University, Burwood, VIC Australia; 11 Department of Medicine, School of Clinical Sciences at Monash Health, Monash University, Clayton, VIC Australia; 12 Institute for Physical Activity and Nutrition, Deakin University, Geelong, Australia; 13 Research Institute of the McGill University Health Centre, Department of Medicine, McGill University, Montreal, QC Canada

**Keywords:** Handgrip strength, Cognitive function, Older adults, CERAD, AFT, DSST

## Abstract

Low handgrip strength, a hallmark measure of whole-body strength, has been linked with greater odds of cognitive decline and dementia; however, conflicting findings, which could be due to population characteristics and choice of tools, such for the assessment of handgrip strength and cognitive function domains, also exist. Therefore, we examined the relationship of handgrip strength with a comprehensive list of tests to assess domains of cognitive function using a representative sample of US older men and women without neurodegenerative disorders such as dementia. We analyzed cross-sectional data from the US National Health and Nutrition Examination Survey (NHANES) between 2011 and 2014, with a study cohort of 777 older adults (380 men and 397 women) above 60 years of age. Handgrip strength was assessed using a handgrip dynamometer, while cognitive function was assessed through the Consortium to Establish a Registry for Alzheimer’s Disease (CERAD) Word List Learning Test (WLLT), Word List Recall Test (WLRT), Intrusion Word Count Test (WLLT-IC and WLRT-IC), the Animal Fluency Test (AFT), and the Digit Symbol Substitution Test (DSST). Sex-stratified multiple linear regression analyses were performed upon covariate adjustment for age, ethnicity, socio-economic status, education, medical history, body mass index, physical activity, energy, protein, and alcohol intake. Maximal handgrip strength was positively associated with cognitive function scores, including CERAD WLLT (*P* = 0.009, *R*^2^ = 0.146) and AFT (*P* = 0.022, *R*^2^ = 0.024) in older men, but not in women (CERAD WLLT: *P* = 0.253, AFT: *P* = 0.370). No significant associations with CERAD WLLRT (men: *P* = 0.057, women: *P* = 0.976), WLLT-IC (men: *P* = 0.671, women: *P* = 0.869), WLLRT-IC (men: *P* = 0.111, women: *P* = 0.861), and DSST (men: *P* = 0.108, women: *P* = 0.091) were observed. Dose–response curves exhibited a prominent linear relationship between all significant associations after covariate adjustment, with no indication of a plateau in these relationships. In conclusion, higher handgrip strength was independently associated with better learning ability for novel verbal information and verbal fluency in US men over the age of 60 and without dementia. Longitudinal studies are required to confirm whether muscle strength independently predicts cognitive function changes in older adults in a sex-specific manner, and whether this connection is affirmed to the possibility of reverse causation due to declines in physical activity levels in the preclinical phase of dementia.

**Supplementary Information:**

The online version contains supplementary material available at 10.1007/s11357-022-00703-3.

## Introduction

Aging is associated with cognitive impairments that lead to deficits in attention, executive function, processing speed, and reaction time [1]. These age-related changes in cognitive function are characterized by alternations in brain structure including gray and white matter shrinkage, decreased dendritic synapses, and neuronal cell death [2]. Interestingly, a concomitant decline in cognitive and physical performance has been observed in older populations as emerging data link low muscle strength with poor cognitive outcomes, including cognitive impairment and dementia [3]. In addition, age-related declines in muscle strength reduce independence and result in poor health outcomes among older adults that are linked to malnutrition, depression, recurrence of falls and fractures, and poor balance [4].

Women appear to be twice as likely to develop dementia than men [5], which is partly due to sex hormone changes after menopause, since hormone therapy is associated with reduced risk of neurodegenerative diseases including dementia [6]. Interestingly, a 12-year longitudinal study revealed a greater number of modifiable risk factors for dementia during middle-to-older age in men compared with women [7]. Loss of physical function may co-occur with losses in cognitive performance as a part of the aging process, but it is not known whether alterations in physical performance correspond with a sex-specific magnitude of cognitive decline. Handgrip strength constitutes a hallmark measure of physical performance and function [8] and it has recently been shown, by using pooled data from general population-based prospective cohort studies, that increased handgrip strength is associated with reduced risk of cognitive impairments such as dementia [3]. Nevertheless, associations between handgrip strength and cognitive outcomes in men and women with good cognitive function tend to be non-significant [3]. Considering that the association between handgrip strength and all-cause mortality is stronger in women than men [9], although the age-related decline in handgrip strength has been speculated to be greater in men compared to women from the age of 30 onwards [10], it is important to investigate sex-specific associations between handgrip strength and health-related and/or functional outcomes.

At present, there is a large body of literature reporting that handgrip strength is linked with multiple aspects of cognition across healthy [11–18], community-dwelling [19], and diseased aging populations [20, 21]. Indeed, lower handgrip strength has been linked with increased white matter hyperintensity volume [22, 23] and lower whole-brain volume [24], both of which are associated with cognitive decline and dementia risk [19, 25, 26].

Sex differences in cognitive function are well documented with women typically achieving higher scores in verbal processing tasks while men in visuospatial processing tasks [27]. Similarly, Ling et al. demonstrated significant cross-sectional associations between handgrip strength and mini-mental state examination (MMSE) scores in older women but not men [28]. By contrast, Kim et al. showed that higher handgrip strength at baseline was positively associated with MMSE scores both in older men and women, but handgrip strength could positively predict MMSE scores over time only in women [29]. Research has also shown a link between handgrip strength and MMSE [30], and Clock Drawing Test performance in both older men and women, but language, short-term, and delayed memory scores only in men [31]. However, considering a potential risk of vascular dementia due to hypertension that is linked to micro- and macro-cerebrovascular lesions, these results may not be applicable to those who are normotensive. Moreover, a previous longitudinal study revealed greater odds of cognitive impairment in older women as opposed to men [32], although that was based on prediction risk rather than the evaluation of a linear response between handgrip strength and aspects of cognition. Finally, handgrip strength has been associated with multiple aspects of cognitive performance such as verbal fluency, sustained attention, processing speed, and working memory in people with history or ongoing incidence of cancer [33]. However, whether handgrip strength is linked with these aspects of cognitive function in a sex-specific manner in community-dwelling older individuals without cognitive impairment remains to be elucidated. Further support for potential sex differences is provided by studies which have shown that testosterone is related to greater handgrip strength in men [34], and free testosterone is associated with significant positive association with processing speed, sustained attention, and working memory in older men above 60 years of age [35]. In addition, previous studies have not controlled for important confounding variables that can influence cognitive function in community-dwelling older adults, such as history of stroke incidence, and arthritis diagnosis [31, 36] or, in some cases, physical activity and nutritional intake [30]. Therefore, there is a need to address these major limitations in order to make concrete conclusions regarding sex-specific differences.

Using a representative national sample of older US adults (≥ 60 years of age) with normal cognitive function from the National Health and Nutrition Examination Survey (NHANES), we comprehensively examined the sex-specific associations between handgrip strength and cognitive function in terms of immediate and delayed learning ability and inhibition for novel verbal information, verbal fluency, sustained attention, processing speed, and working memory.

## Methods

### Study design and participants

Cross-sectional data was retrieved from participants aged ≥ 60 years with normal cognitive function using the NHANES between 2011 and 2014. NHANES is a project conducted by the Centers for Disease Control and Prevention (CDC) and the National Center for Health Statistics (NCHS) that is comprised of interviews (demographic, socioeconomic, dietary, and medical history-related questions), physical examinations (anthropometrical, medical, and physiological measurements), and biochemical analyses. The NHANES protocol is approved by the NCHS Research Ethics Review Board and all participants provided written informed consent.

### Handgrip strength assessment

Handgrip strength was assessed using a handgrip dynamometer (Takei Digital 5401) by a trained examiner that was monitored by field supervisors. A practice trial was utilized to determine if participants understood the procedure, which was followed by a repeated dynamometer squeeze from each hand with the participant in a seated position. Each hand was tested three times with a 60-s rest between the same hand measurements. Handgrip strength was measured in kilograms (kg) and expressed as the maximum value out of the three handgrip attempts in each hand.

### Cognitive assessment

Cognitive function was assessed in terms of immediate and delayed learning ability and inhibition for novel verbal information—Consortium to Establish a Registry for Alzheimer’s Disease (CERAD) Word List Learning Test (WLLT), Word List Recall Test (WLRT), and Intrusion Word Count Test (WLLT-IC and WLRT-IC)—, verbal fluency [Animal Fluency Test (AFT)], and sustained attention, processing speed, and working memory [Digit Symbol Substitution Test (DSST)]. Scoring in the CERAD WLLT, WLLT-IC, WLRT, and WLRT-IC ranged between 0 and 10, in AFT from 1 to 40, and in DSST from 0 to 100. Higher test scores represented better cognitive performance. Participants that did not provide complete data for any of the aforementioned cognitive function tests were excluded from the study. These tests have been previously validated for use in research practice.

### Covariates

Age (years), ethnicity (race), socioeconomic status (family income to poverty ratio (FIPR)), education level (school qualification), medical history [stroke incidence and arthritis (osteoarthritis, rheumatoid arthritis, psoriatic arthritis) diagnosis], body mass index (BMI) (kg/m^2^), physical activity (minutes), daily energy intake (kcal), protein (g), and alcohol intake (g) were used as covariates. All covariates were potential confounders in the relationship between handgrip strength and cognitive performance.

Age groups consisted of participants aged ≥ 60 years and classified per decade into 60–69, 70–79, and ≥ 80 years of age to distinguish potential changes that may occur in each decade. Ethnic groups were comprised of Mexican American, other Hispanic, non-Hispanic White, non-Hispanic Black, non-Hispanic Asian, and other (multi)races. Social economic status was categorized as low-middle [family income to poverty ratio (FIPR) < 1] and middle-high (FIPR ≥ 1). Education level was defined as no high school degree, at most a high school degree, or a college degree at minimum. Medical history in terms of loss of cognitive-memory function, stroke incidence, and arthritis diagnosis was categorized as Yes/No responses based on past incidence reported by a doctor or other health professional. Physical activity was expressed as minutes of moderate-intensity recreational activities on a typical week. A physical activity of < 150 min per week was considered as low-moderate and ≥ 150 min as moderate-high. A BMI < 18 kg/m^2^ was considered as low, 18–24.9 kg/m^2^ as moderate, and ≥ 25 kg/m^2^ as high. Energy, protein, and alcohol intake were calculated as averages of the 24-h recall and categorized into low, moderate, and high. In men, energy intake < 2000 kcal was considered as low, 2000–3000 kcal as moderate, and > 3000 kcal as high. In women, energy intake of < 1600 kcal was considered as low, 1600–2400 kcal as moderate, and > 2400 kcal as high. Protein intake ≤ 0.8 g/kg/body weight was considered low, and > 0.8 g/kg/body as high. In men, alcohol intake < 15 g was considered as low, 15–30 g as moderate, and > 30 g as high. In women, alcohol intake < 10 g was considered as low, 10–20 g as moderate, and > 20 g as high.

### Statistical analysis

Multiple linear regression analyses were performed to examine the association between handgrip strength and cognitive function in terms of test-specific cognitive performance by sex with adjustment of all covariates. A restricted cubic spline was employed to model the non-linear and dose–response relationship between handgrip strength and cognitive function using three knots after covariate adjustments. Handgrip strength and cognitive performance were considered as continuous variables, and all covariates were categorical in type. Statistical significance was established as *P* < 0.05 and statistical analysis was performed using IBM SPSS Statistics v28.

## Results

### Characteristics of study participants

Handgrip strength and cognitive performance data were available for 777 participants (380 men and 397 women). Baseline sociodemographic, anthropometric, nutritional, and medical history characteristics stratified by sex are summarized in Tables S1 and S2.

Most men were between 60 and 69 years of age (55.8%). These were mostly non-Hispanic White (52.1%) and Black (21.7%), of middle-high socioeconomic status (91.6%) with a college degree at minimum (65.8%). Energy intake was mostly lower than the recommended value (50.3%) or just within this range (42.6%). For most of the participants, protein intake was higher than the recommended value (66.8%). Alcohol intake was low (78.4%) and BMI was high (70.0%). Most participants had moderate-high physical activity (85%). Twenty (5.3%) participants had a history of stroke and 134 (35.3%) had an arthritis diagnosis. The average handgrip strength in men was 40.1 (± 0.4) kg.

Most women were between 60 and 69 years of age (60.2%). These were mostly non-Hispanic White (52.4%) and Black (22.2%), of middle-high socioeconomic status (88.9%) with a college degree at minimum (63.7%). Energy intake was mostly lower than the recommended value (48.4%) or just within this range (44.6%). For most of the participants, protein intake was higher than the recommended value (58.7%). Alcohol intake was low (83.8%) and BMI was high (71.5%). Most participants had moderate-high physical activity (84.1%). Seventeen (4.3%) participants had a history of stroke and 210 (52.9%) had an arthritis diagnosis. The average handgrip strength in women was 25.4 (± 0.2) kg.

### Handgrip strength and cognitive function

**Fig. 1:**
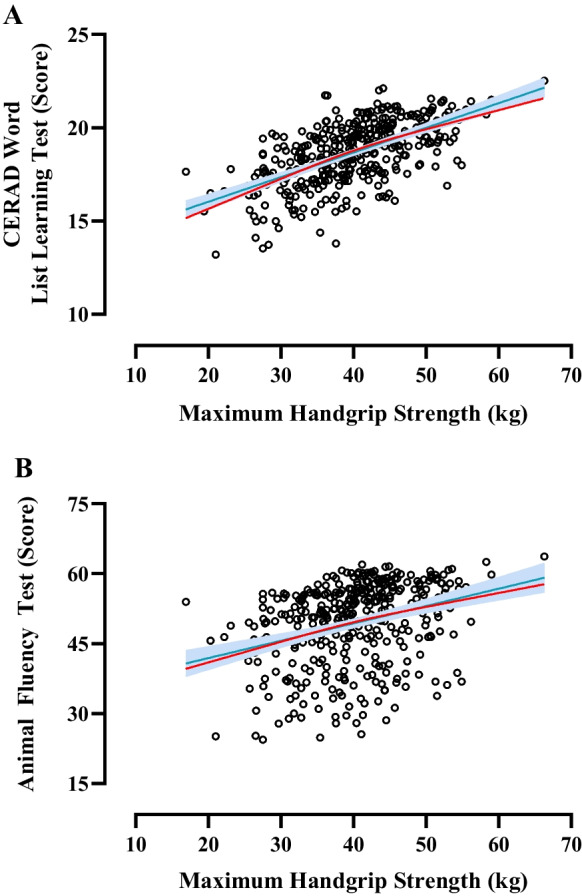
Dose–response relationship between handgrip strength and cognitive function in male participants. Significant associations with CERAD WLLT (**A**) and AFT (**B**) scores were observed. Linear (blue) and spline (red) models were adjusted for age, gender, ethnicity, socio-economic status, educational level, and medical history (stroke incidence and arthritis diagnosis, body mass index, physical activity, alcohol, protein, and energy intake). Abbreviations: AFT Animal Fluency Test, CERAD Consortium to Establish a Registry for Alzheimer’s Disease, WLLT Word List Learning Test

Handgrip strength was positively associated with CERAD WLLT (*P* = 0.009) and AFT (*P* = 0.022) in older men but not in women (CERAD WLLT: *P* = 0.253, AFT: *P* = 0.370). No associations were found between handgrip strength and CERAD WLLRT (men: *P* = 0.057, women: *P* = 0.976), WLLT-IC (men: *P* = 0.671, women: *P* = 0.869), WLLRT-IC (men: *P* = 0.111, women: *P* = 0.861), and DSST (men: *P* = 0.108, women: *P* = 0.091) (Table 1). Dose–response curves displayed a linear relationship between all significant associations after adjustment for covariates, with no indication of a plateau in these relationships (Fig. 1). Similar analysis of the total cohort showed significant associations with CERAD WLLT (unadjusted *P*: 0.008; adjusted *P*: 0.003) and WLLRT (unadjusted *P*: 0.033), AFT (unadjusted *P*: 0.004; adjusted *P*: 0.010), and DSST (adjusted *P*: 0.025) (Table S3).

**Table 1:** Sex-stratified multiple linear regression analysis of the association between maximum handgrip strength and cognitive function by sex, upon covariate adjustment for age, ethnicity, socio-economic status, education, medical history (stroke incidence and arthritis diagnosis), BMI, physical activity, energy, and alcohol intake

Cognitive tests	Men			Women		
	*β*	*P*	*R*^2^	*β*	*P*	*R*^2^
CERAD WLLT	0.084	0.009	0.146	0.050	0.253	0.178
CERAD WLLRT	0.030	0.057	0.133	− 0.001	0.976	0.146
CERAD WLLT-IC	0.004	0.671	0.047	0.002	0.869	0.052
CERAD WLLRT-IC	− 0.009	0.111	0.041	− 0.001	0.861	0.056
AFT	0.087	0.022	0.024	0.049	0.370	0.234
DSST	0.157	0.108	0.334	0.273	0.091	0.349

*AFT* Animal Fluency Test, *BMI* body mass index, *CERAD* Consortium to Establish a Registry for Alzheimer’s Disease, *DSST* Digit Symbol Substitution Test, *WLLT* Word List Learning Test, *WLRT* Word List Recall Test. *WLLT-IC* Word List Learning Test—Intrusion Word Count, *WLRT-IC* Word List Recall Test—Intrusion Word Count

## Discussion

In older adults aged 60 years and above living in the USA, handgrip strength was significantly positively associated with learning ability for novel verbal information and verbal fluency in older men but not in women, suggesting that future research is needed to unravel the neurobiological and physiological mechanisms that underpin the differences in both handgrip strength and cognitive function declines during aging in a sex-specific manner.

Our findings are in line with previous research showing sex-specific discrepancies in associations between handgrip strength and domains of cognitive function. Indeed, in a recent study in a cohort of Chinese hypertensive older participants (~ 60 years), higher handgrip strength was linked with increased language, short-term, and delayed memory scores in older men (assessed via Digit Span Forward and Backward Test, Verbal Fluency Test, and Verbal Recall Test), but no associations were observed in older women [31]. However, the authors did not control for important variables that may alter sex-specific responses to indices of cognitive function, including physical activity, energy, and nutrient intake. On the other hand, in cancer survivors (breast, prostate, colon, and cervix cancer) above 60 years of age, handgrip strength was associated with verbal fluency (AFT), sustained attention, processing speed, and working memory (DSST) in both men and women [33]. It is possible that disease states and the degree to which a patient is exposed may overcome the potential sex-specific associations between handgrip strength and cognitive function at an older age, since in our relatively physically active population, associations were observed with a verbal fluency test only in men.

Our results also conflict with prior longitudinal studies on the topic. For instance, in a 7-year cohort of Mexican Americans aged over 65 years, lower handgrip strength was correlated with reductions in MMSE scores, a test which assesses multiple cognitive domains, in both sexes [30]. Likewise, a 4.4-year follow-up also failed to identify a sex-specific relationship between handgrip strength and MMSE scores in both older men and women 72 years of age, while in another 8-year longitudinal cohort of Korean adults aged 65 years or older, correlations between handgrip strength and cognitive function (via K-MMSE score) were observed in both sexes [37]. Moreover, no sex-specific differences have been found between men and women in the oldest old. Specifically, in a 4-year longitudinal cohort of older Dutch adults aged 89 years, reduced handgrip strength was associated with lower MMSE scores in both men and women [28], while another 1-year longitudinal sample of older Italian men and women 80 years of age showed a significant association between handgrip strength and Clock Drawing Test (CDT) in both sexes [38]. The aforementioned findings from longitudinal studies reveal that studying the relationship of handgrip strength with cognitive domains such as MMSE and CDT infers identical results in both older men and women. However, considering that MMSE is a widely used instrument relying on socioeconomic and educational characteristics as well as a mixture of cognitive aspects [39], the potential sex-specific differences may be more relevant to specific cognitive domains rather than composite scores of multiple tests [36]. Our findings also suggest that strength may be more relevant to cognitive domains (i.e., verbal processing tasks) that men typically perform poorer in than women [27].

Based on the current scarcity of the literature, the mechanisms contributing to differential sex-specific associations between handgrip strength and distinct domains of cognitive function among observational studies are unclear. In a 16-year longitudinal British cohort of adults aged 69–71 years, a stronger association between handgrip strength and higher whole-brain volume at follow-up was observed in women compared to men [12]. Interestingly, bioavailable testosterone may be a link mediating this relationship in men, considering its associations with indices of cognitive function highlighted in this study [35]. Nevertheless, further insights on how handgrip strength may alter brain physiology under conditions of sexual dimorphism and how that translates to sex-specific alterations in cognitive function are warranted. However, sampling bias should also be taken into account in relation to the aforementioned findings pertaining to sex-specific differences. Considering the cross-sectional data collection and the lack of gold-standard assessment tools for overall cognitive function, it is worth stating that one-time measurement of exposure and outcome does not support causal relationships [40]. Ignoring sampling variance may lead to misinterpretation of underlying biological pathways that we have previously described in the above sections [41]. For example, sampling error due to convenience sampling referring to variations in participant willingness to take part in some of the assessments tests in such studies with large sample sizes may partially explain our results [42, 43]. Given the limited mechanistic evidence that could explain the sex-specific differences found in handgrip strength and its association with indices of cognitive function, these are plausible factors that should be considered.

### Strengths and limitations

Our study employed nationally representative data that has undergone rigorous quality control, and multiple confounders were adjusted to accurately estimate the association between handgrip strength and several domains of cognitive function in older men and women without dementia. However, this study was also prone to limitations. Observational studies using data from cross-sectional surveys are unable to exhibit a causal relationship between dependent and independent variables. In addition, NHANES uses specific tests of cognitive performance that may not fully represent overall cognitive function. Cognitive function is comprised of multiple interconnected mental processes and hence, more refined assessment tools are required. For instance, backward number recall involves greater attention and executive function reserves compared to forward recall testing [44]. In addition, data availability regarding handgrip strength and cognitive performance from NHANES was limited to only two annual cycles, which could amplify sampling bias. Finally, poor physical capacity may rather be a consequence of cognitive decline during aging rather than its cause. This may be of particular importance in longitudinal studies that have not appropriately controlled for physical activity throughout the longitudinal period. The likelihood of reverse causality should also not be dismissed since physical activity levels are reduced before the clinical diagnosis of dementia [45] while previous research has also linked higher white matter hyperintensities with lower physical performance in older populations [46].

## Conclusions

Handgrip strength is positively associated with learning ability for novel verbal information and verbal fluency in US older men aged 60 years and above, but not in older women. There is also scope for future studies to confirm the role of strength as a risk factor for decline in specific domains of cognitive function, and specifically in domains where men typically perform worse than women; this can then follow with promotion of appropriate interventions for maintenance and enhancement of strength as a potential means for attenuation of age-related cognitive decline. However, our findings should be interpreted with caution due to their cross-sectional nature. Future studies are warranted to uncover the biological mechanisms to confirm the directionality and explore the potential sex-specific relationship between handgrip strength and components of cognitive function in older men and women, while exercise interventions could unravel potential links involving these factors.

## Supplementary Information

Below is the link to the electronic supplementary material.

Supplementary file1 (DOCX 26 KB)
