# Supplementary material for: Handgrip strength is associated with learning and verbal fluency in older men without dementia: insights from the NHANES
Source: GeroScience. 2022 Nov 30;45(2):1049–58. doi: 10.1007/s11357-022-00703-3 (PMC9886698; doi:10.1007/s11357-022-00703-3)
Supplement: Supplementary file 1 — Supplementary file1 (DOCX 26 KB) [file 11357_2022_703_MOESM1_ESM.docx]

**Supplementary Material**

| **Table S1.** Socio-demographic, behavioural and nutritional characteristics of male participants (*n* = 380). Values are expressed as count (percentage) unless otherwise specified. | |
| --- | --- |
| **Age** |  |
| 60-69 years | 212 (55.8) |
| 70-79 years | 115 (30.3) |
| ≥ 80 years | 53 (13.9) |
| **Ethnicity** |  |
| Mexican American | 31 (8.2) |
| Other Hispanic | 27 (7.1) |
| Non-Hispanic White | 198 (52.1) |
| Non-Hispanic Black | 84 (22.1) |
| Non-Hispanic Asian | 33 (8.7) |
| Other Race - Including Multi-Racial | 7 (1.8) |
| **Socio-economic status** |  |
| Low-Middle | 32 (8.4) |
| Middle-High | 348 (91.6) |
| **Educational level** |  |
| No high school degree | 59 (15.5) |
| High school degree | 71 (18.7) |
| College degree | 250 (65.8) |
| **Energy intake** |  |
| Low | 191 (50.3) |
| Moderate | 162 (42.6) |
| High | 27 (7.1) |
| **Protein intake** |  |
| Low | 126 (33.2) |
| High | 254 (66.8) |
| **Body mass index** |  |
| Low | 4 (1.1) |
| Normal | 110 (28.9) |
| High | 266 (70.0) |
| **Alcohol intake** |  |
| Low | 298 (78.4) |
| Moderate | 40 (10.5) |
| High | 42 (11.1) |
| **Physical activity** |  |
| Low-Moderate | 57 (15.0) |
| Moderate-High | 323 (85) |
| **Medical history** |  |
| Stroke | 20 (5.3) |
| Arthritis | 134 (35.3) |
| **Handgrip strength (kg)** |  |
| Minimum | 16.9 |
| Average^*^ | 40.1 (7.7) |
| Maximum | 66.3 |
| * Values expressed as mean (standard deviation) | |

| **Table S2.** Socio-demographic, behavioural and nutritional characteristics of female participants (*n* = 397). Values are expressed as count (percentage) unless otherwise specified. | |
| --- | --- |
| **Age** |  |
| 60-69 years | 239 (60.2) |
| 70-79 years | 109 (27.5) |
| ≥ 80 years | 49 (12.3) |
| **Ethnicity** |  |
| Mexican American | 30 (7.6) |
| Other Hispanic | 37 (9.3) |
| Non-Hispanic White | 208 (52.4) |
| Non-Hispanic Black | 88 (22.2) |
| Non-Hispanic Asian | 31 (7.8) |
| Other Race - Including Multi-Racial | 3 (0.8) |
| **Socio-economic status** |  |
| Low-Middle | 44 (11.1) |
| Middle-High | 353 (88.9) |
| **Educational level** |  |
| No high school degree | 53 (13.4) |
| High school degree | 91 (22.9) |
| College degree | 253 (63.7) |
| **Energy intake** |  |
| Low | 192 (48.4) |
| Moderate | 177 (44.6) |
| High | 28 (7.1) |
| **Protein intake** |  |
| Low | 164 (41.3) |
| High | 233 (58.7) |
| **Body mass index** |  |
| Low | 3 (0.8) |
| Normal | 110 (27.7) |
| High | 284 (71.5) |
| **Alcohol intake** |  |
| Low | 333 (83.9) |
| Moderate | 29 (7.3) |
| High | 35 (8.8) |
| **Physical activity** |  |
| Low-Moderate | 63 (15.9) |
| Moderate-High | 334 (84.1) |
| **Medical history** |  |
| Stroke | 17 (4.3) |
| Arthritis | 210 (52.9) |
| **Handgrip strength (kg)** |  |
| Minimum | 8.2 |
| Average^*^ | 25.4 (5.0) |
| Maximum | 40.6 |
| * Values expressed as mean (standard deviation) | |

| **Table S3.** Simple (unadjusted) and multiple linear regression analysis of the association between maximum handgrip strength and cognitive function by sex, upon covariate adjustment for age, sex, ethnicity, socio-economic status, education, medical history (stroke incidence and arthritis diagnosis), BMI, physical activity, energy, and alcohol intake | | | | | | |
| --- | --- | --- | --- | --- | --- | --- |
|  | **Unadjusted** | | | **Adjusted** | | |
| **Cognitive Tests** | **β** | ***P*** | ***R*^2^** | **β** | ***P*** | ***R*^2^** |
| CERAD WLLT | -0.043 | 0.008 | 0.009 | 0.074 | 0.003 | 0.205 |
| CERAD WLLRT | -0.017 | 0.033 | 0.006 | 0.018 | 0.146 | 0.167 |
| CERAD WLLT-IC | 0.001 | 0.821 | 0.000 | 0.004 | 0.542 | 0.041 |
| CERAD WLLRT-IC | -0.001 | 0.724 | 0.000 | -0.006 | 0.106 | 0.040 |
| AFT | 0.056 | 0.004 | 0.010 | 0.080 | 0.010 | 0.206 |
| DSST | -0.030 | 0.619 | 0.000 | 0.187 | 0.025 | 0.353 |
| **Abbreviations**: AFT: Animal Fluency Test, BMI: Body Mass Index, CERAD: Consortium to Establish a Registry for Alzheimer's Disease, DSST: Digit Symbol Substitution Test, WLLT: Word List Learning Test, WLRT: Word List Recall Test. WLLT-IC: Word List Learning Test – Intrusion Word Count, WLRT-IC: Word List Recall Test – Intrusion Word Count. | | | | | | |
